# A systematic review of syphilis serological treatment outcomes in HIV-infected and HIV-uninfected persons: rethinking the significance of serological non-responsiveness and the serofast state after therapy

**DOI:** 10.1186/s12879-015-1209-0

**Published:** 2015-10-28

**Authors:** Arlene C. Seña, Xiao-Hui Zhang, Trudy Li, He-Ping Zheng, Bin Yang, Li-Gang Yang, Juan C. Salazar, Myron S. Cohen, M. Anthony Moody, Justin D. Radolf, Joseph D. Tucker

**Affiliations:** Department of Medicine, Institute for Global Health and Infectious Diseases, University of North Carolina at Chapel Hill, Chapel Hill, NC USA; Sexually Transmitted Diseases Department, Guangdong Provincial Dermatology Hospital, Guangzhou, China; School of Medicine, University of North Carolina at Chapel Hill, Chapel Hill, NC USA; Department of Pediatrics, Division of Pediatric Infectious Diseases, University of Connecticut and Connecticut Children’s Medical Center, Farmington, Connecticut USA; Department of Pediatrics, Division of Pediatric Infectious Diseases, Duke University, Durham, North Carolina USA; Duke Human Vaccine Institute, Duke University, Durham, North Carolina USA; Department of Medicine, UConn Health, Farmington, Connecticut USA

**Keywords:** Syphilis, Systematic review, Serologic non-response, Serofast, Treatment response

## Abstract

**Background:**

Syphilis remains a global public health threat and can lead to severe complications. In addition to resolution of clinical manifestations, a reduction in nontreponemal antibody titers after treatment is regarded as “proof of cure.” However, some patients manifest < 4-fold decline (“serological non-response”) or persistently positive nontreponemal titers despite an appropriate decline (“serofast”) that may represent treatment failure, reinfection, or a benign immune response. To delineate these treatment phenomena, we conducted a systematic review of the literature regarding serological outcomes and associated factors among HIV-infected and -uninfected subjects.

**Methods:**

Six databases (PubMed, Embase, CINAHL, Web of Science, Scopus, and BIOSIS) were searched with no date restrictions. Relevant articles that evaluated serological treatment responses and correlates of serological cure (≥ four-fold decline in nontreponemal titers) were included.

**Results:**

We identified 1693 reports in the literature, of which 20 studies met selection criteria. The median proportion of patients who had serological non-response was 12.1 % overall (interquartile range, 4.9–25.6), but varied depending on the time points after therapy. The serofast proportion could only be estimated from 2 studies, which ranged from 35.2–44.4 %. Serological cure was primarily associated with younger age, higher baseline nontreponemal titers, and earlier syphilis stage. The relationship between serological cure and HIV status was inconsistent; among HIV-infected patients, CD4 count and HIV viral load was not associated with serological cure.

**Conclusions:**

Serological non-response and the serofast state are common syphilis treatment outcomes, highlighting the importance of determining the immunological and clinical significance of persistent nontreponemal antibody titers after therapy.

**Electronic supplementary material:**

The online version of this article (doi:10.1186/s12879-015-1209-0) contains supplementary material, which is available to authorized users.

## Background

Syphilis is an ancient infectious disease, yet modern efforts to control the disease remain ineffective in many countries, especially among high risk populations like men who have sex with men (MSM) [1–3]. The World Health Organization (WHO) estimated that in 2008, there were 36 million prevalent cases of syphilis and 11 million incident cases in adults between the ages of 15 and 49 [4]. Although the majority of cases have occurred in underdeveloped regions like Sub-Saharan Africa, countries like China and Russia have witnessed alarming increases in syphilis rates attributed in part to societal and economic changes [5, 6]. A significant association exists between syphilis and increased risk of HIV acquisition, with hazard ratios ranging from 2.3 to 8.6 [7–9]. A recent review of studies conducted worldwide reported a 9.5 % prevalence of syphilis among adults living with HIV infection [10].

Syphilis has been associated with protean clinical manifestations, making reliance upon laboratory diagnosis critical for the practicing physician. However, due to our inability to cultivate the causative agent of syphilis *in vitro,* most testing for syphilis relies on measurement of immune responses rather than on direct tests for *Treponema pallidum* [11]. Direct detection methods are available for early syphilitic lesions, but polymerase chain reaction (PCR) assays have limited sensitivities for detecting spirochetes from blood, especially during latent infection [12, 13]. Testing for syphilis involves serological assays based on nontreponemal and treponemal antibody responses induced by *T. pallidum.* Nontreponemal tests measure IgM and IgG antibodies to lipoidal antigens, principally cardiolipin, released from damaged host cells and/or *T. pallidum* [11, 14]. Nontreponemal antibody tests are still primarily used for syphilis screening in the United States (U.S.) and developing countries, and to monitor serological response to treatment since titers are observed to decline after effective therapy. Treponemal assays, on the other hand, measure specific IgM and IgG antibodies to *T. pallidum* proteins and have been traditionally utilized to confirm reactive nontreponemal tests.

Since 1993, U.S. treatment guidelines have regarded a four-fold (or two dilution; e.g., 1:64 to 1:16) decline in nontreponemal antibody titers or seroreversion to negative as an indicator of an appropriate serological response after treatment [15, 16]. This recommendation was based on a study by Brown, et al. [17], in which the investigators generated curves demonstrating a four-fold decline in Venereal Disease Research Laboratory (VDRL) titers at three months following therapy, using data from patients who had symptom resolution after treatment of primary (PS) and secondary syphilis (SS). However, some patients in clinical practice do not follow the classical patterns of serological response to therapy, exhibiting less than a four-fold decline in nontreponemal titers and/or persistently low positive titers without evidence of treatment failure or reinfection. These conditions have been referred to as “serological failure,” “serological non-response,” “seroresistance,” “reagin-fast,” or the “serofast state.”

In addition to the lack of consensus regarding the terminology used to refer to nontreponemal antibody titers that do not meet exhibit an appropriate serological response after therapy, there is also uncertainty regarding whether these conditions indicate persistent infection or a residual immune response in the absence of viable organisms. Although retreatment may be beneficial for some of these patients, there are no specific markers that can differentiate those requiring additional therapy to prevent subsequent sequelae. Untreated or inadequately treated syphilis can result in severe neurological and cardiovascular complications, stillbirth, neonatal death, and irreversible congenital abnormalities [18, 19]. Not surprisingly, considerable controversy exists whether patients who do not demonstrate an appropriate serological treatment response should undergo lumbar punctures to evaluate for neurosyphilis or be retreated.

A large number of studies have been conducted to investigate serological responses after treatment of syphilis in HIV-infected and HIV-uninfected individuals. Therefore, we performed a systematic review to evaluate the frequency of serological outcomes following syphilis therapy and correlates associated with serological cure. This review illustrates the extensive observational data that have accrued regarding lack of serological cure after treatment, and highlights the need for more definitive studies regarding the immunological and clinical significance of persistent nontreponemal antibodies over time. We hypothesize that these persistent antibodies are associated with a failure of immune tolerance rather than lack of pathogen clearance after recommended therapies.

## Methods

### Search strategy and selection criteria

A systematic review of the literature was conducted on original research articles using the terms “syphilis” and “serology” and “failure”, “resistance”, “response”, “serofast”, or “seroresistance” in PubMed, Embase, Cumulative Index to Nursing and Allied Health, Web of Science, Scopus, and BIOSIS with no date restrictions. In addition, the metaRegister of controlled trials (www.controlled-trials.com) and the U.S. National Institutes of Health Ongoing Trials Register (www.clinicaltrials.gov) were searched for ongoing clinical trials. The search was last updated on November 14, 2014. In addition, syphilis experts were contacted to identify other potential manuscripts. Authors of relevant articles were contacted for further information as needed. This literature review was registered in PROSPERO (CRD42013005016). The search was conducted according to Preferred Reporting Items for Systematic Reviews and Meta-Analyses (PRISMA) guidelines. The full search strategy and PRISMA checklist are included as Additional file 1.

### Study selection

**Fig. 1:**
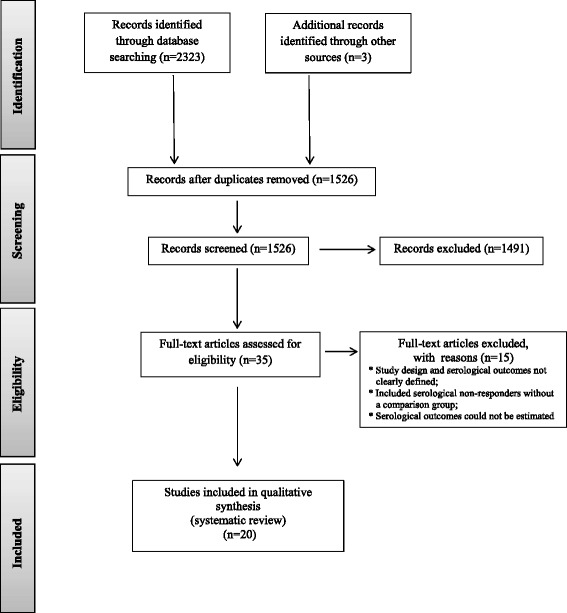
PRISMA flow diagram of the systematic review process using the terms “syphilis” and “serology” and “failure”, “resistance”, “response”, “serofast”, or “seroresistance” in six databases

A database search was performed for English language research articles, reports, abstracts, and clinical trials related to the serological outcome of syphilis treatment in adults. Articles were first examined and selections were made based on titles. The abstracts of these articles were then examined for relevance to the outcomes of interest and full text articles were reviewed. The full citation screening process is detailed using the PRISMA flow diagram in Fig. 1.

### Eligibility criteria, data abstraction, and analysis

Articles that defined and evaluated serological outcomes after syphilis therapy among HIV-infected and/or -uninfected patients were included in the review. Studies were excluded if study design and serological outcomes were not clearly defined in the methods, if the study only included serological non-responders without a comparison group, or if serological outcomes could not be estimated in the results due to combined categories (e.g. serological non-responders with treatment failures or reinfections).

Serological cure was defined as a ≥ four-fold decline (at least two titers, such as 1:64 to 1:16) in nontreponemal titers or seroreversion to nonreactive results after therapy. The presence of persistent nontreponemal antibody titers were delineated into two categories distinct from treatment failure or reinfection: (1) “serological non-response” defined as < four-fold decline in nontreponemal antibody titers at ≥ six months after treatment for early syphilis or ≥ 12 months following treatment of late latent syphilis, OR (2) “serofast status” defined as persistently positive low-level nontreponemal antibody titers without seroreversion after initial ≥ 4-fold decline. Treatment failure was defined as a ≥ four-fold rise in nontreponemal titers after treatment in the absence of reinfection. Reinfection was defined as a ≥ four-fold rise in nontreponemal titers after treatment, supported by a history of having had unprotected sex with a potentially infected person. The primary outcome was the proportion of study subjects identified with serological non-response at 6 and ≥ 12 months from therapy and clinical correlates of serological cure. Secondary outcomes were the proportions reported as serofast, treatment failures or reinfections, and correlates associated with serological response.

The following characteristics of each study were abstracted: study period, sample size, study population, study location, study design, percentage of males and MSM, prior history of syphilis, HIV prevalence, syphilis stages, and neurosyphilis prevalence based on cerebrospinal fluid (CSF) analysis. Data abstraction was also performed for the following information on diagnosis, treatment, and serological outcomes: type of nontreponemal antibody test; main and alternative treatment regimens; proportion with serological cure, serological non-response, serofast state, treatment failure, and reinfection; and correlates of serological cure. Data abstraction was performed twice independently and there was >95 % concordance. Conflicting data were resolved by a third independent individual.

A qualitative synthesis of the data was performed, and the median and interquartile range for serofast frequency was calculated. Meta-analysis was not feasible due to heterogeneity in the reporting of serological outcomes.

### Quality assessment

The STROBE checklist [20] was used to assess the quality of the observational studies included in our literature review. These evaluations are included in the Additional file 2: Table S1.

## Results

### Study characteristics

We identified 1526 unduplicated articles in the literature and reviewed 291 abstracts. Twenty articles were included in our systematic review (Fig. 1), of which 12 were retrospective studies [21–32] and eight were prospective in design [33–40]. The retrospective studies were based on review of clinical records from patients for whom baseline and follow-up nontreponemal antibody titers were available. The prospective studies included five observational studies [33–35, 39, 40], and three randomized controlled trials (RCTs) [36–38].

The characteristics of the study populations from these articles are summarized in Table 1. The age of study subjects ranged from 18–71 years; one study involved only women [33] while 12 studies included >50 % men. In four studies, the majority (>94 %) of subjects were identified as MSM [25, 32, 39, 40]. The studies involved patients from 13 diverse countries worldwide (Table 1). Three of the prospective investigations were multicenter studies, each of which included eight separate clinical sites for enrollment [37, 38, 40].

**Table 1:** Study design and characteristics of the study population among 20 studies included in the systematic review

First author	Study design	Study time period	Study location	Number of study subjects	Study population	Male sex (%)	Men who have sex with men (%)	HIV prevalence (%)	Primary syphilis (%)	Secondary syphilis (%)	Early latent syphilis (%)	Late latent syphilis (%)	Tertiary or neuro-syphilis (%)
Dionne-Odom	R cohort	2002–2008	Lusaka, Zambia and Kigali, Rwanda	1321	HIV-serodiscordant couples	40	NR	70.6	NR			NR	NR
Fiumara	R cohort	NR	Boston, Massachusetts (US)	128	University hospitals	64	NR	NR	0	0	0	100	NR
Ghanem (2007) [23]	R cohort	1992–2000	Baltimore, Maryland (US)	297	STD clinic	44	NR	43.4	62.6			37.4	0
Ghanem^a^ (2008) [24]	R cohort	1990–2006	Baltimore, Maryland (US)	180	University HIV clinic	67.2	NR	100	26.8^b^			55.4^b^	17.8^b^
Goemen^b^	P cohort	NR	Kinshasa, Zaire	193	Women’s health center	0	0	37	NR			NR	NR
Jinno	R cohort	2006–2011	Los Angeles, California (US)	560	Urban HIV clinics	99	96.7	100	14	26	60	0	0
Knaute^c^	R cohort	1999–2008	Zurich, Switzerland	264	University and general hospitals	92	NR	42	34	50	13^d^		3
Li	R cohort	2000–2010	Beijing, China	501	University hospital	50.9	NR	0	17.4	51.1	31.5	0	0
Long^e^	P cohort	2003	Lima, Peru	96	Neighborhood venues	NR	NR	15.6	NR			NR	NR
Malone^f^	R cohort	1985–1991	San Diego, California (US)	100	Naval Medical Center	99^e^	NR	100	3.6	16.1	17.9	53.6	8.9
Manavi^g^	P cohort	2003–2005	Birmingham UK	261	STD clinics	76^f^	38.3	38.3	0	0	100	0	0.4
Reidner	P RCT	2000–2003	Myeba Region, Tanzania	328	High risk populations	28.4	NR	52.1	7.6	0	92.8	0	0
Rolfs	P RCT	1991–1994	NR	541	NR	70.8	12.8	18.7	25.7	46.8	27.5	0	HIV+: 61 %
													HIV-: 40 %
Romanowski	R cohort	1981–1987	Alberta, Canada	882	Provincial public health records	17	21.1	NR	68.3	18.3	4.3	0	NR
Seña	P RCT	2000–2009	Birmingham New Orleans, Los Angeles, Baltimore, Durham, Raleigh, Indianapolis, (US); Antana-narivo Tamatave, Madagascar	465	STD clinics	61.7	3.0	0	24.7	46.9	28.4	0	0
Tittes^h^	R cohort	1987–2010	Vienna, Austria	249	University STD-outpatient clinic	83.1		31.7	26.1	73.9	0	0	0
Tong	R cohort	2005–2010	Xiamen, China	1327	University hospital	58.9	NR	0	22.0	27.1	31.4^i^		19.5^d^
Tsai	R cohort	2007–2013	Taiwan	394	HIV care hospitals	100	94.9	100	8.9	55.3	35.8	0	0
Wu^j^	P cohort	2009–2013	Taiwan	174	University hospital	99.7	97.0	100	17.6	53.7	28.7	0	0
Yang	P cohort	2007–2012	Taiwan	573	HIV care hospitals	NR	94.1	100	8.9	57.8	33.3	0	0

*R* retrospective, *P* prospective, *RCT* randomized controlled trial, *NR* not reported

^a^ Demographics and serological outcome data were reported for 180 patients; however, syphilis stages were reported for 231 cases of syphilis

^b^ Demographics were reported for 193 patients; however, serological outcome data were reported for 120 patients

^c^ Demographics and syphilis stages were reported for 264 patients; however, serological outcome data were reported for 214 patients

^d^ This value is the total percentage of latent syphilis patients. This study did not distinguish between early latent and late latent patients

^e^ Demographics were reported for 96 patients; however, serological outcome data were reported for 76 patients

^f^ Demographics and syphilis stages were reported for 100 patients; however, serological outcome data were reported for 56 patients

^g^ Demographics and syphilis stages were reported for 371 patients; however, serological outcome data were reported for 261 patients

^h^ Demographics and syphilis stages were reported for 379 patients; however, serological outcome data were reported for 249 patients

^i^ This value is the total percentage of latent syphilis patients. This study did not distinguish between early latent and late latent patients

^j^ Demographics and syphilis stages were reported for 296 patients; however, serological outcome data were reported for 174 patients

**Table 2:** Syphilis treatment regimens, nontreponemal antibody tests, estimated proportions with serological cure and serological non-response at time points after therapy

First Author	Main syphilis treatment regimens	Alternative syphilis treatment regimens	Non-treponemal antibody test	% Serological cure (≥ 4-fold decline in nontreponemal titers or seroreversion)	% Serological non-response (< 4-fold decline in nontreponemal titers)	Time period assessed after therapy
Dionne-Odom	1–3 doses benzathine PCN G (2.4 MU IM weekly)	None	RPR	66.8	26.6	400 days
Fiumara	2 doses benzathine PCN G (2.4 MU IM weekly)	Tetracycline	RPR	64.1	33.6	< 5 years
Ghanem (2007) [23]	Benzathine PCN G (1 dose for early syphilis, 3 doses for late syphilis)	Doxycycline	RPR	87.9	9.1	400 days
Ghanem (2008) [24]	Benzathine PCN G (1 dose for early syphilis, 3 doses for late syphilis)	Doxycycline	RPR	60.5	14.4	9–12 months^a^
Goemen	3 doses benzathine PCN G (2.4 MU IM weekly)	None	RPR	23.4	71.3	6 months
				27.5	62.5	12 months
				40.0	56.7	24 months
Jinno	1–3 doses benzathine PCN G (2.4 MU IM weekly)	Doxycycline	RPR	90.9	9.1	9–12 months
Knaute	1–3 doses benzathine PCN G (2.4 MU IM weekly); aqueous PCN G for tertiary syphilis	None	VDRL	96.3	3.7	6 months
				97.7	2.3	9 months
				99.1	0.9	12–18 months
Li	2 doses benzathine PCN G (2.4 MU IM weekly)	Not reported	RPR	85.0	15.0	6 months
Long	1–3 doses benzathine PCN G (2.4 MU IM weekly)	Doxycycline	RPR	93.4	6.6	12 months
Malone	3 doses benzathine PCN G (2.4 MU IM weekly)	PCN G procaine with probenecid or IV PCN G	VDRL	79.8	2.3	12 months
Manavi	1–3 doses benzathine PCN G (2.4 MU IM weekly)	Doxycycline for 21 days	VDRL	68.2	31.4	12 months
Reidner	1 dose benzathine PCN (2.4 MU IM)	Azithromycin	RPR	96.4	3.6	9 months
Rolfs	1 dose benzathine PCN (2.4 MU IM)	1 dose benzathine PCN enhanced with 10 day course of amoxicillin and probenecid	RPR	89.3	10.7	6 months
				90.3	9.7	12 months
Romanowski	1 dose benzathine PCN (2.4 MU IM)	Tetracycline or erythromycin	RPR	77.9	22.1	6 months
				88.8	11.2	12 months
				95.1	4.9	24 months
Seña	1 dose benzathine PCN (2.4 MU IM) or azithromycin (2g PO)	Doxycycline	RPR	78.5	20.5	6 months
Tittes	1–3 doses benzathine PCN (2.4 MU IM weekly)	None	VDRL	95.2	4.8	6 months
Tong	1–3 doses benzathine PCN (2.4 MU IM weekly)	Azithromycin IM or IV	RPR	64.1	35.9	6 months
				65.6	34.4	12 months
Tsai	1 dose benzathine PCN (2.4MU IM)	Doxycycline	RPR	69.5	25.9	6 months
				67.8	32.3	12 months
Wu	1 dose benzathine PCN or azithromycin	3 doses of benzathine PCN or doxycycline	RPR	74.7	25.3	6 months
Yang	1–3 doses benzathine PCN (doses NR)	None	RPR	79.1	5.6	6 months
				70.9	4.2	12 months

*PCN* penicillin, *MU* million units, *IM* intramuscular, *IV* intravenous, *RPR* rapid plasma reagin, *VDRL* venereal disease research laboratory; *NR* not reported

^a^ Serological outcomes were determined at ≥ 9 months after treatment for early syphilis or ≥ 12 months for late syphilis

Ten studies enrolled only subjects with early syphilis (ES) [25, 27, 29, 30, 32, 36–40], which corresponded to PS, SS, or early latent syphilis (ELS); one study evaluated only subjects with late latent syphilis (LLS) [22]. Six studies involved subjects with LLS, tertiary syphilis and/or neurosyphilis at time of treatment [22–24, 26, 28, 35, 37], and three did not specify subjects’ stage of syphilis in their study [21, 33, 34]. Two of the six studies [24, 37] classified neurosyphilis in their patients based on CSF markers including an elevated protein, white blood cell count and/or a reactive CSF VDRL assay, while the others used only a reactive CSF VDRL or did not specify the CSF criteria for diagnosis.

Fifteen studies included HIV-infected patients with syphilis in their analyses, of which six focused entirely on the serological response to syphilis therapy among study subjects with HIV infection [24, 25, 28, 32, 39, 40]. Subjects from the studies which included only HIV-infected patients had mean CD4 counts ranging from 280–565 cells/μL and mean plasma viral loads ranging from 2.21–3.10 log_10_ copies/mL [32, 39]; their median HIV RNA levels ranged from < 400 to 8742 copies/mL [24, 25]. The proportion of HIV-infected patients who were on antiretroviral therapy (ART) ranged from 60–87 % [25, 40].

Among the 20 studies, 2.4 million units of benzathine penicillin (PCN) G administered intramuscularly once or weekly for three weeks was reported as the main treatment regimen (Table 2). However, several alternative treatment regimens were provided to study subjects, including azithromycin, doxycycline, erythromycin, tetracycline and other PCN formulations. The RCT by Rolfs, et. al. [37] randomized ES patients to receive 2.4 million units of benzathine PCN with or without an enhanced regimen consisting of a 10 day course of amoxicillin and probenecid.

Most of the studies used the Rapid Plasma Reagin (RPR) as the nontreponemal test to monitor serological response. There were, however, variations in the length of follow-up after treatment ranging from six months to five years (Table 2). One retrospective study reported a median follow-up time of 5.3 years [24]. For the prospective studies, retention rates for study subjects ranged from 61–94 % at six months and 52–81 % at 12 months after enrollment [36–40].

### Definitions and time points after therapy

Although all of the studies defined an appropriate serological response as a ≥ four-fold decline in nontreponemal antibody titers after treatment, there were differences in the terminology used to refer to a < 4-fold decline in nontreponemal titers following therapy. Several investigators referred to or grouped these outcomes with treatment failures (i.e. a 4-fold rise in titers) [23, 24, 28, 32, 35, 37]. Four studies used the term “serofast state” to refer to serological non-responders [21, 27, 30, 38]. Accordingly, we redefined the data and results from these studies using our criteria for serological outcomes (see Methods).

There were differing time points after therapy at which serological outcomes were determined, but which were generally consistent with clinical guidelines for monitoring [41]. Four studies reported only on the proportion of ES patients who met serological outcomes at six months [27, 28, 38, 39]. The other studies determined their outcomes at varied time points for other stages of infection (e.g. nine-12 months, 12 months, 400 days). The retrospective study by Romanowski, et al. [29] analyzed the most time points after treatment (i.e., at six, 12, and 24 months) for changes in RPR titers from baseline among 1090 ES patients.

### Frequency of serological outcomes after therapy

Among the 20 studies included in our review, the median proportion of patients who had serological non-response after treatment was 12.1 % overall (interquartile range [IQR]: 4.9 – 25.6). At six months, the median proportion of patients who were serological non-responders following therapy was 20.5 % (IQR: 8.2 - 25.6). This proportion decreased to 11.2 % (IQR: 5.8 – 31.9) at ≥ 12 months for all stages of syphilis. Using data from the 10 studies that included only ES patients, the median proportion of serological non-response was 9.4 % (IQR: 4.8 – 19.1); only one study by Fiumara [22] was restricted to LLS patients, for which the proportion with serological non-response was estimated at 21.1 %. Very few studies in our review provided estimates of the proportion of patients who had a ≥ 4-fold decline in nontreponemal antibody titers after treatment but remained serofast over time; the estimated proportion was 35.2 % in the study by Fiumara [22] among patients with LLS, and 44.4 % in the cohort reported by Ghanem, et al. [24] among HIV-infected patients with both ES and late syphilis [24].

Five studies reported treatment failures, which met the definitions for a ≥ four-fold rise in nontreponemal titers after treatment in the absence of reinfection; the proportion reported from these studies ranged from 0–24 % [23, 24, 28, 33, 37]. Ghanem, et al. [24] reported the highest proportion of treatment failures among 44/180 of HIV-infected patients; however, the investigators acknowledged the difficulty in distinguishing between treatment failures and reinfections in the absence of behavioral or network data. Seven studies reported reinfections among their syphilis patients, with estimates ranging from 0.2–10 % [21, 23, 26, 32, 35, 37, 40].

### Association of serological outcomes with patient demographics

Ten studies assessed the association of patient age with serological response to therapy (Table 3). Five found that younger age was significantly associated with serological cure [24, 30–32, 38], and the other five reported no association [25, 29, 35, 39]. In a retrospective study of 1327 HIV-negative persons, Tong, et al. [31] found that patients < 23 years of age had a 2.2 adjusted odds ratio (AOR) (95 % CI: 1.1–4.2) for serological cure compared to those > 40 years. Tsai et al. [32] demonstrated that HIV-infected patients aged < 34 who received benzathine PCN were twice as likely to achieve serological cure at 6 months after treatment compared to older HIV-infected individuals. Regarding gender, only two of five studies found a significant association with serological outcomes. Tong et al. [31] reported that serological cure was associated with male sex, while Dionne-Odom, et al. [21] noted an association with female sex.

**Table 3:** Patient characteristics and other factors evaluated for their associations with serological outcomes after syphilis therapy

First Author	PatientAge	Patient Gender	Baseline nontrepo-nemal titers	Stage of syphilis infection	History of prior syphilis	Trepo-nemal IgM	Syphilis treatment regimen	HIV coinfection	CD4 cell count	HIV viral load	Antiretroviral therapy
Dionne-Odom		+	+	+				—			
Fiumara			+								
Ghanem (2007) [23]				—				+			
Goemen								—			
Knaute				+				—			
Li			+	+		+					
Long			+				—	—			
Manavi	—		+		+	—		—			
Reidner	—	—	+	+			—	—			
Rolfs					—		—	+			
Romanowski	—	—	+	+	+						
Seña	+		+	+	—		—				
Tittes	+	—	+	—			+	—			
Tong	+	+	+	+							
Ghanem (2008) [24]	+						—	H	+	—	+
Jinno	—		+	—	+		—	H	+	—	—
Malone								H	—		
Tsai	+				—		—	H	—	—	—
Wu	—		—	+				H	—	—	—
Yang			+	+	—		+	H	—	—	—

H indicates that the study included HIV-infected participants only

+ indicates a significant association in multivariate analyses between the variable and serological outcomes after therapy

– indicates that a significant association was not identified between the variable and serological outcomes after therapy

### Association of serological outcomes with baseline nontreponemal antibody titers

Thirteen studies evaluated the association of baseline nontreponemal antibody titers with serological treatment response. Of these, ten found that higher baseline titers (e.g., ≥ 1:32) were significantly associated with a greater likelihood of or time to serological cure in both HIV-infected and HIV-uninfected patients (Table 3) [21, 23, 25, 27, 29–31, 34–36, 38]. Tong, et al. [31] reported that a baseline titer ≤ 1∶2 or ≥ 1∶64 was associated with an increased likelihood of serological cure among patients with PS, SS, latent and tertiary syphilis. In contrast, Romanowski, et al. [29] reported that lower baseline titers (e.g. ≤ 1:8) in ES were associated with an increased likelihood of seroreversion. One other study found no association between baseline nontreponemal titers and subsequent serological outcomes [39].

### Association of serological outcomes with stage of syphilis

Twelve studies evaluated the association of syphilis stage with serological outcomes, of which seven observed an association between earlier stage of infection and cure [21, 26, 27, 29, 31, 36, 38–40]. In general, there was an increased likelihood of or time to serological cure among patients with PS or SS compared to those with ELS. Wu, et al. [39] reported that HIV-infected patients with ELS had a decreased AOR of 0.32 (95 % CI 0.14–0.71) for serological cure at 6 months after therapy compared to patients with PS or SS. Six studies included patients with later stages of syphilis but had limited data [22–24, 26, 28, 31]; Tong, et al. [31] did report that patients with ES had a higher likelihood of a serological cure at 12 months (OR 2.4) compared to patients with late syphilis.

### Association of serological outcomes with treatment regimen

Nine studies evaluated the effect of treatment regimens on serological outcomes, but only two found any significant associations. Yang, et. al. [40] reported an increased AOR of 1.68 (95 % CI 1.20–2.36) for serological cure with three weekly doses of benzathine PCN compared to one dose among HIV-infected patients with ES. However, Tittes, et al. [30] noted higher serological cure rates overall with one dose of benzathine PCN compared to three doses of weekly PCN (98 % vs. 92 %, *p* = 0.003) among both HIV-infected and HIV-uninfected patients with PS or SS. In this study, serological cure rates among HIV-infected patients were reported to be 88 % with one PCN dose compared to 97 % with three doses, but the difference was not found to be statistically significant (*p* = 0.18) [30].

### Association of serological outcomes with HIV status

Nine studies evaluated the association of HIV status with serological outcomes after treatment of syphilis by comparing patients with and without HIV infection. Of these studies, only two reported that HIV-infected patients were less likely to achieve serological cure [24, 37]; the other seven indicated that HIV status did not affect treatment outcomes [21, 26, 29, 33–36]. Ghanem, et al. [23] noted that HIV-infected patients with ES had a significantly increased risk of serological failure, and that those with LLS had a slower response time to treatment than HIV-uninfected patients (342 vs 138 days respectively, *p* = 0.03). The RCT by Rolfs, et. al. [37] which included 101 HIV-infected persons reported that patients with PS had an AOR of 7.6 (95 % CI: 1.3–44.2) for serologic treatment failure at six months compared to patients without HIV infection. Furthermore, multivariate analysis of the mean decrease in the RPR titer showed that PS patients co-infected with HIV had a significantly slower titer decrease than HIV-uninfected patients. The latter study is one of few to measure the rate of reduction of nontreponemal antibody titers over time. However, the differences between HIV-infected and HIV-uninfected persons were not found to be statistically significant for patients who had SS or ELS [37].

### Factors associated with serological outcomes among HIV-infected persons with syphilis

Six studies analyzed the serological response after treatment solely among HIV-infected persons (Table 3). Ghanem, et al. [24] reported that a CD4 cell count of < 200 cells/mL was associated with an increased risk of serological failure (adjusted hazard ratio, 2.5; 95 % CI: 1.3–4.9), and that use of ART was associated with a 60 % reduction in the rate of serological failure. Jinno, et al. [25] similarly noted that a lower CD4 count (i.e. < 350 cells/mL) among HIV-infected patients was significantly associated with serological failure after treatment. However, other studies found no association between CD4 count, HIV viral load, or ART among participants with HIV infection [28, 30, 39, 40].

### Other factors and their association with serological outcomes

Additional factors have been evaluated for their associations with serological outcomes following syphilis treatment (Table 3). Seven studies assessed a prior history of syphilis, of which three noted a significant association with treatment response. Two of the studies demonstrated a lower likelihood of cure among patients with prior syphilis infection [25, 29]; however, Manavi et al. [35] found that past syphilis treatment was significantly associated with serological response to current therapy. Two studies noted conflicting correlations between the detection of treponemal IgM antibodies in patients’ serum and serological treatment response [27, 35], which is a marker for early infection.

### Lumbar punctures among patients with serologic non-response

Only one study performed lumbar punctures among individuals with serological non-response to therapy [22]. Fiumara evaluated the CSF in six patients who did not exhibit a four-fold decrease in nontreponemal titers after treatment, including one individual that had a RPR titer of 1:32 for four years. None of the patients that he evaluated were found to have reactive spinal fluids suggestive of neurosyphilis [22].

## Discussion

*T. pallidum* cannot be cultured *in vitro*, thus forcing reliance on serological tests for diagnosis and proof of cure. This practice has been in place for nearly a century due to the absence of novel technologies to confirm eradication of the organism. Unfortunately, our systematic review of 20 studies conducted worldwide among HIV-infected and HIV-uninfected patients with syphilis demonstrates that a substantial proportion (12.1 %) of patients exhibit serological non-response after treatment, which is time-dependent. The median proportion of serological non-response patients was 20.5 % at six months, which decreased to 11.2 % at ≥ 12 months for all stages of syphilis. Our findings highlight the importance of developing a consistent definition of serological non-response, which is not an absolute or definite condition but should be thought of as a continuum with serological cure and the serofast state over time. The presence of persistent nontreponemal antibodies may represent treatment failure, reinfection, or another undefined immune phenomenon; however, none of the studies in our review could distinguish among these possibilities when neither a four-fold rise in nontreponemal antibody titers nor a history of re-exposure was evident. The appropriate time to determine falling titers, and the correlates that affect the time when titers fall, remains unclear. Serological cure was less likely in older individuals, patients with lower baseline nontreponemal antibody titers, and patients with later stages of syphilis. The clinical or biological explanations for these observations have not been well established. Perhaps more importantly, there are no consistent criteria by which patients can be stratified to determine their need for additional evaluation or syphilis therapy when serological non-response exists.

Nontreponemal antibodies have played a pivotal role in syphilis screening and monitoring of treatment outcomes; yet the mechanism by which they arise is poorly understood. The first serological test for syphilis was developed in 1906 as a complement fixation assay by Wasserman,et. al. [42], using antigens from human and monkey extracts rich in *T. pallidum.* Other investigators soon discovered that antibodies from syphilis patients reacted not just with antigens from spirochetes but also with cardiolipin found in normal nonsyphilitic tissues. Pangborn subsequently purified cardiolipin from beef heart extract [43], which in combination with lecithin and cholesterol, serves as the antigen for current nontreponemal antibody tests. The central question is whether the cardiolipin antibodies detected in syphilis patients are a response specific to the spirochete, since the lipid composition of purified *T. pallidum* has been identified as 13 % cardiolipin [14, 44]. However, individuals who have no other evidence for syphilis infection have been found to have anticardiolipin antibodies similar to those detected in syphilitic patients, leading to the hypothesis that this class of antibodies may be generated in response to destruction of host tissues [45]. Nontreponemal antibodies have been observed in persons with a variety of infectious and autoimmune diseases including HIV, hepatitis, and systemic lupus erythematosus [46–48], and these potentially false positive tests continue to cause problems in diagnosis and management.

The possibility that persistent nontreponemal antibodies after treatment represent persistence of *T. pallidum* was raised by early investigators, based on findings from animal and human studies conducted in the 1960s [49, 50]. These studies identified treponemes in lymph nodes and CSF of rabbits one year after therapy for LLS, and reported similar findings in human subjects [49]. However, subsequent investigations questioned whether these treponemes were viable, since the transfer of material from the lymph nodes of some treated syphilis patients produced no detectable disease upon inoculation into rabbits [50]. Studies of the natural history of syphilis have demonstrated that *T. pallidum* can disseminate to multiple organs in humans within hours of inoculation [46]. *T. pallidum* is then known to infect various anatomical sites, including the central nervous system and eye as “immune privileged” areas where the spirochetes can slowly replicate and evade the immune response [46]. Symptomatic and asymptomatic neurosyphilis has been reported in a case series of serofast patients previously treated for ES [51]; however, the study involved only a small number of patients and is contrary to the findings by Fiumara [22].

In order to address the question of persisting spirochetes after therapy, highly sensitive direct detection methods for *T. pallidum* would be essential for analyzing patient specimens before and after treatment. Unfortunately, a meta-analysis of studies evaluating the performance of *T. pallidum* PCR on samples taken during different stages of infection reported pooled sensitivities as low as 31.2 % and 47.4 % in blood and CSF specimens, respectively [52], illustrating their limitations in ruling out residual spirochetes in previously treated patients. In the absence of a readily available test of cure based on detection of the organism, there is no other definitive method to detect persistent *T. pallidum* in serological non-responders or serofast patients. This suggests that other means, such as analysis of antigen-specific immune responses that occur during infection and after therapy, may be a more pragmatic approach to investigating these conditions.

The hypothesis that a lingering immune response is responsible for persistent nontreponemal antibodies can be argued based on a number of key observations. We found that 11 % of patients still exhibit serological non-response at ≥ 12 months after therapy; yet reported tertiary syphilis cases are still relatively uncommon worldwide. Thus, the observed proportion of serological non-response is not likely to be associated with a high frequency of cardiovascular or neurological complications as might be expected, given the global rate of syphilis. PCN has been considered to be highly effective since it was first reported for treatment of syphilis [53]. *T. pallidum* is extremely sensitive to minute amounts of PCN *in vitro* and *in vivo* if maintained for sufficient periods of time [54, 55], and presently, there are no data to suggest that the spirochete has developed PCN resistance. Furthermore, there are no clear benefits to providing additional therapy for serological non-responders or serofast patients although they may repeatedly undergo retreatment in clinical practice. A recent study involving ES patients who had serological non-response after initial therapy and received an additional dose of benzathine PCN demonstrated that 73 % still failed to have an appropriate serological response and only 2 % seroreverted at 12 months despite retreatment [56]. These findings imply that persistent nontreponemal antibody titers may not be due to insufficient therapy, but rather an alternative process like the immune response to treponemal infection. Presently, there have been no other studies demonstrating that retreatment of patients who are serological non-responders leads to improved outcomes.

Treponemal IgM and IgG antibodies have already been demonstrated to persist in syphilis patients after therapy [57–59], indicating the presence of plasma cells that continue to produce *T. pallidum* specific antibodies. This kind of pathogen-specific memory response is seen after both infections and vaccinations. Long-term study of human volunteers has shown that pathogen-specific memory responses have very slow decay rates with apparent half-lives ranging from years to millennia [60]. Therefore, the persistence of treponemal antibodies is expected despite clearance of infection after treatment.

In contrast, nontreponemal antibodies decline after therapy in the majority of syphilis patients, indicating that they are down-regulated after clearance of *T. pallidum*. These antibodies are autoreactive since cardiolipin, the main antigenic component that binds to nontreponemal antibodies, is present in mitochondrial membranes. Disappearance of these autoreactive antibodies after syphilis therapy leading to seroreversion suggests the involvement of tolerance control. In serological non-response and the serofast state, there is an apparent failure of the expected process for down-regulation. Persisting nontreponemal antibodies after effective therapy may thus represent failure of immune tolerance rather than lack of pathogen clearance. Recent work on HIV has shown that many antibodies against the virus are autoreactive and under tolerance controls [61–67], and careful study of these antibodies has provided clues to their origin and regulation. Understanding the nature of nontreponemal antibodies in syphilis patients who do not have an appropriate serological response could shed light on their origins, and allow differentiation between antibodies indicative of persistent infection or mere antibody persistence in the absence of infection.

Our review found conflicting evidence on the relationship between demographic, clinical factors, HIV status and serological response. The associations of syphilis stage and baseline nontreponemal antibody titers with serological treatment outcomes appear to be the most consistent among the studies in this review. Only two out of nine studies reported that HIV-infected status decreased the likelihood and time to serological cure [23, 37]. Markers of immune function such as CD4 count and HIV viral load also do not appear to affect serological outcomes among HIV-infected persons with syphilis [28, 32, 39, 40]. Patients with HIV infection have well described dysfunction of antibody formation [68], and the former finding may be due to this B cell dysfunction [69] in which there is less effective immune clearance of *T. pallidum* in HIV-infected persons. The epidemiological connection between HIV and syphilis is well-established [70, 71], but an understanding of the biological and immunological relationships between these co-infections remains obscure.

Several limitations are evident from this systematic review. First, there was no standard definition for serological non-response that was consistent. There was heterogeneity in the classifications of serological outcomes that did not meet the criteria for serological cure, which is partly related to the lack of consensus of the U.S. and European Union guideline definitions [41, 72]. This discordance presented challenges in the overall synthesis of results, and may have affected our estimates. Secondly, there were variations in follow-up times when a patient was considered to have serological non-response after treatment. Longer follow-up periods are necessary in order to determine serological outcomes over time; however, this must be balanced by the challenges of having patients return for serological monitoring years after completion of therapy. In general, most of the studies defined ELS as syphilis acquired < 1 year prior according to U.S. and European CDC criteria rather than < 2 years [41, 72]; however, there is a potential that some LLS cases were included as early cases resulting in lower response rates due to non-discriminatory misclassification. Only a few studies in our review provided data on the serofast state, treatment failures or reinfections; therefore, our estimates about the frequency of these conditions were limited. Finally, there has only been a handful of prospective studies to assess serological outcomes following treatment of syphilis. The majority of the studies reviewed were retrospective in study design, which presents disadvantages due to the potential for selection bias and misclassification as a result of non-standardized data collection and follow-up periods after treatment.

Our results highlight a fundamental problem in the management of *T. pallidum* infections that has prevailed over decades. Clinicians have been taught to expect at least a four-fold decline in nontreponemal antibody titers or seroreversion to following therapy; however, serological non-response occurs in nearly one out of every eight patients with syphilis after treatment. This significant proportion and our clinical observations regarding serological non-response and the serofast state suggest the following plausible scenarios that patients may eventually exhibit serological response or serorevert over time (e.g. as in LLS); patients may be getting reinfected at a high rate; patients may not be achieving 100 % cure from PCN; or that patients with persistent nontreponemal antibody titers have neither reinfection nor persistent *T. pallidum* but instead, an altered immune process in which their anticardiolipin antibodies are not down-regulated. Given the diversity of data presented here, it is conceivable that each of these possibilities holds true for a subset of patients, and we presently lack sufficient information to differentiate among these or other scenarios. There have been vast improvements in methods for detection of microorganisms, and in understanding the host response to different infections. Unfortunately, modern innovative technologies have not yet been applied to the study of syphilis. Based on the data from our systematic review, we do not believe that further retrospective studies of serological response to recommended therapies will be useful or informative. Rather, coordinated investigations involving novel B cell methods, direct detection assays for *T. pallidum*, and correlations with nontreponemal and treponemal antibody tests in syphilis-infected patients are essential to advancing clinical management of this formidable pathogen.

## Conclusions

Syphilis remains a global public health threat, whose clinical management is highly dependent on nontreponemal antibody titers. Unfortunately, the basis for the cardiolipin antibodies detected during syphilis infection is poorly understood, and our systematic review affirms that a significant proportion of both HIV-infected and HIV-uninfected patients will have serological non-response after therapy. These patients may have reinfection, treatment failure, or a benign immune response. In order to explore these possibilities and inform clinical practice, efforts must be directed towards designing prospective syphilis studies and utilizing modern technologies to determine the microbiological and immunological basis for these antibodies.

## Additional file

Additional file 1: Table S1. STROBE assessment. Checklist from the Strengthening the Reporting of Observational Studies in Epidemiology (STROBE) Initiative of items that were reported or not reported in the 20 studies included in our review. (DOCX 28 kb) Additional file 2: Table S2. PRISMA checklist. The Preferred Reporting Items for Systematic Reviews and Meta-Analyses (PRISMA) Statement checklist of 27 items pertaining to the content of our systematic review.(DOC 64 kb)

## Abbreviations

MSM: Men who have sex with men (MSM)
ES: Early syphilis
PS: Primary syphilis
SS: Secondary syphilis
ELS: Early latent syphilis
LLS: Late latent syphilis
VDRL: Venereal Disease Research Laboratory
CSF: cerebrospinal fluid
ARV: antiretroviral therapy
RPR: Rapid Plasma Reagin
PCR: polymerase chain reaction
AOR: adjusted odds ratio
OR: odds ratio
