# Supplementary material for: A systematic review of syphilis serological treatment outcomes in HIV-infected and HIV-uninfected persons: rethinking the significance of serological non-responsiveness and the serofast state after therapy
Source: BMC Infect Dis. 2015 Oct 28;15:479. doi: 10.1186/s12879-015-1209-0 (PMC4625448; doi:10.1186/s12879-015-1209-0)
Supplement: Additional file 1: Table S1. — STROBE assessment. Checklist from the Strengthening the Reporting of Observational Studies in Epidemiology (STROBE) Initiative of items that were reported or not reported in the 20 studies included in our review. (DOCX 28 kb) [file 12879_2015_1209_MOESM1_ESM.docx]

**Supplementary Content**

Search Statements

1. Embase

syphilis:de,ab,ti AND (serolog*:de,ab,ti AND (fail*:de,ab,ti OR resist*:de,ab,ti OR response*:de,ab,ti) OR serofast*:de,ab,ti OR seroresistance:de,ab,ti)

2. CINAHL

((MH "Syphilis+") OR syphilis) AND ((serolog* AND (fail* OR resist* OR response*)) OR serofast OR seroresistance)

3. Web of Science

(syphilis AND ((serolog* AND (fail* OR resist* OR response*)) OR serofast OR seroresistance))

4. BIOSIS

(syphilis AND ((serolog* AND (fail* OR resist* OR response*)) OR serofast OR seroresistance))

5. PubMed

(Syphilis[mesh] OR syphilis[tw]) AND ((serolog*[tw] AND (fail*[tw]OR resist*[tw] OR response*[tw])) OR serofast[tw] OR seroresistance[tw])

6. Scopus

(syphilis AND ((serolog* AND (fail* OR resist* OR response*)) OR serofast OR seroresistance))

**Supplementary Table 1. STROBE Assessment**

| **STROBE Items** | **Dionne-Odom** | **Fiumara** | **Ghanem 2007** | **Ghanem 2008** | **Goeman** | **Knaute** | **Li** | **Long** | **Malone** | **Rieder** | **Rolfs** | **Romanow-ski** | **Sena** | **Tong** | **Manavi** | **Yang** | **Jinno** | **Tsai** | **Tittes** | **Wu** |
| --- | --- | --- | --- | --- | --- | --- | --- | --- | --- | --- | --- | --- | --- | --- | --- | --- | --- | --- | --- | --- |
| Indicate the study’s design with a commonly used term in the title or abstract | R | NR | R | R | R | R | NR | R | R | NR | R | NR | R | R | R | R | R | R | R | NR |
| Provide in the abstract an informative and balanced summary of what was done and what was found | R | R | R | R | R | R | NA | R | R | R | R | R | R | R | R | R | R | R | R | R |
| Explain the scientific background and rationale for the investigation being reported | R | R | R | R | R | R | R | R | R | R | R | R | R | R | R | R | R | R | R | R |
| State specific objectives, including any pre-specific hypotheses | R | R | R | R | R | R | R | R | R | R | R | R | R | R | R | R | R | R | R | R |
| Present key elements of the study design early in the paper | R | R | R | R | R | R | R | R | R | R | R | R | R | R | R | R | R | R | R | R |
| Describe the setting, locations, and relevant dates, including periods of recruitment, exposure, follow-up, and data collection | R | NR | R | R | R | R | R | R | R | R | R | R | R | R | R | R | R | R | R | R |
| Give the eligibility criteria, and the sources and methods for selection of participants | R | R | R | R | R | R | R | R | R | R | R | R | R | R | R | R | R | R | R | R |
| For matched studies, give matching criteria and number of exposed and unexposed | NA | NA | NA | NA | NA | NA | NA | NA | NA | NA | NA | NA | NA | NA | NA | NA | NA | NA | NA | NA |
| Clearly define all outcomes, exposures, predictors, potential confounders, and effect modifiers. Give diagnostic criteria, if applicable | R | NR | R | R | R | R | R | NR | R | R | R | R | R | R | R | R | R | R | R | R |
| For each variable of interest, give sources of data and details of methods of assessment (measurement). Describe comparability of assessment methods if there is more than one group | R | R | R | R | R | R | R | R | R | R | R | R | R | R | R | R | R | R | R | R |
| Describe any efforts to address potential sources of bias | R | NR | R | R | R | R | NR | NR | NR | R | R | R | R | R | NR | R | R | R | R | NR |
| Explain how the study size was arrived at | R | R | R | R | R | R | R | R | R | R | R | R | R | R | R | R | R | R | R | R |
| Explain how quantitative variables were handled in the analyses. If applicable, describe which groupings were chosen and why | R | R | R | R | R | R | R | R | R | R | R | R | R | R | R | R | R | R | R | R |
| Describe all statistical methods, including those used to control for confounding | R | NR | R | R | R | R | R | NR | NR | R | R | R | R | R | R | R | R | R | R | R |
| Describe any methods used to examine subgroups and interactions | R | NR | R | R | R | R | R | NR | R | R | R | R | R | R | R | R | R | R | R | R |
| Explain how missing data were addressed | R | NR | NR | NR | R | R | NR | NR | NR | R | R | R | NR | NR | NR | R | R | R | R | NR |
| If applicable, describe analytical methods taking account of sampling strategy | NA | NA | NA | NA | NA | NA | NA | NA | NA | NA | NA | NA | NA | NA | NA | NA | NA | NA | NA | NA |
| Describe any sensitivity analyses | NA | NA | NA | NA | NA | NA | NA | NA | NA | NA | NA | NA | NA | NA | NA | NA | NA | NA | NA | NA |
| Report numbers of individuals at each stage of study – e.g., numbers potentially eligible, confirmed eligible, included, followed up, and analyzed | R | R | R | R | R | R | NR | NR | R | R | R | R | R | R | R | R | R | R | R | NR |
| Give reasons for non-participation at each stage | R | R | R | R | R | R | NR | NR | R | R | NR | R | R | R | R | R | R | R | R | NR |
| Consider use of a flow diagram | R | NR | NR | NR | NR | NR | NR | NR | NR | R | NR | NR | R | R | NR | R | NR | NR | R | NR |
| Give characteristics of study participant (e.g., demographic, clinical, social) and information on exposures and potential confounders | R | R | R | R | R | R | R | NR | R | R | R | R | R | R | R | R | R | R | R | R |
| Indicate number of participants with missing data for each variable of interest | R | R | R | R | R | R | NR | R | R | R | NR | R | R | R | R | R | R | R | R | NR |
| Report numbers of outcome events or summary measures | R | R | R | R | R | R | R | R | R | R | R | R | R | R | R | R | R | R | R | R |
| Give unadjusted estimates and, if applicable, confounder-adjusted estimates and their precision (eg 95% CI). Make clear which confounders were adjusted for and why they were included | R | NR | R | R | R | R | NR | R | R | R | R | R | R | R | R | R | R | R | R | R |
| Report category boundaries when continuous variables were categorized | NR | R | R | R | R | R | R | R | R | R | R | R | R | R | R | R | R | R | R | R |
| If relevant, consider translating estimates of relative risks into absolute risks for a meaningful time period | NA | NA | NA | NA | NA | NA | NA | NA | NA | NA | NA | NA | NA | NA | NA | NA | NA | NA | NA | NA |
| Report other analyses done – eg, analyses of subgroups and interactions, and sensitivity analyses | R | NR | R | R | R | R | R | R | R | R | R | R | R | R | R | R | R | R | R | R |
| Summarize key results with reference to study objectives | R | R | R | R | R | R | NR | R | R | R | R | R | R | R | R | R | R | R | R | R |
| Discuss limitations of the study, taking into account sources of potential bias or imprecision. Discuss both direction and magnitude of any potential bias | R | R | R | R | NR | R | NR | R | NR | R | R | NR | R | R | R | R | R | R | R | R |
| Give a cautious overall interpretation of results considering objectives, limitations, multiplicity of analyses, results from similar studies, and other relevant evidence | R | R | R | R | R | R | NR | R | R | R | R | R | R | R | R | R | R | R | R | R |
| Discuss the generalizability (external validity) of the study results | R | R | R | R | R | R | NR | NR | R | R | R | R | R | R | R | R | R | R | R | R |
| Give the source of the funding and the role of the funders for the present study and, if applicable, for the original study on which the present article is based. | R | NR | R | R | R | NR | NR | R | R | R | NR | R | R | R | NR | R | NR | NR | R | R |
| Summary of items 1-33 | 28/29 | 18/  29 | 27/  29 | 27  /29 | 27/  29 | 27/  29 | 15  /28 | 19/  29 | 24/  29 | 28/  29 | 25/  29 | 27/  29 | 28/  29 | 28/  29 | 25/  29 | 29/  29 | 27  /29 | 27/  29 | 29/  29 | 22/  29 |
